# Variability in Stigma Severity During the COVID-19 Pandemic

**DOI:** 10.7759/cureus.46508

**Published:** 2023-10-05

**Authors:** İrfan Esen, Selda Kaya, Ersin Günay, Duygu Özol

**Affiliations:** 1 Internal Medicine, Yüksek İhtisas University, Ankara, TUR; 2 Pulmonary Diseases, Yüksek İhtisas University, Ankara, TUR; 3 Pulmonary Diseases, Ankara Etlik City Hospital, Ankara, TUR; 4 Pulmonary Diseases, Süreyyapaşa Chest Diseases And Thoracic Surgery Training And Research Hospital, Ankara, TUR

**Keywords:** social stigma, sars-cov-2 infection, pandemic, stigmatization, covid-19

## Abstract

Objective

The aim of this study was to investigate change in the stigma that emerged during the COVID-19 pandemic over time and the factors responsible for the change.

Methods

Individuals with COVID-19 who presented to Ankara Medicalpark and VM Medicalpark Hospitals' Internal Diseases and Chest Diseases polyclinic between May 2021 and April 2022 were examined. The volunteers were divided into two groups: those who had COVID-19 within the first six months of the pandemic (group 1) and those who had it in the second six months (group 2). The questionnaire assessing stigma consisted of 29 propositions that participants could mark whether they agreed with them or not.

Results

The median age of the volunteers was 38 years. Eighty-eight (69.3%) had the disease in the first six months of the pandemic and 39 (30.7%) in the second six months. Moreover, 76.1% of the participants in the first group and 94.9% of those in the second group did not agree with the statement "I thought COVID-19 was a punishment for me" (p=0.011). Further, 56.8% of the participants in the first group and 97.4% of those in the second group stated that they did not agree with the statement "Employers may terminate the employment of employees who they find out have contracted COVID-19" (p<0.001). 80.7% of the participants in the first group and 38.5% of those in the second group agreed with the statement "There was social discrimination against people who caught COVID-19" (p<0.001).

Conclusions

At the beginning of the pandemic, the participants had concerns about losing their status and jobs, but this anxiety decreased over time. Stigma in the first six months of the pandemic was greater than that in the second six months, and discrimination related to stigma decreased with recognition of the disease and the increase in experience.

## Introduction

Stigma is defined as a quality that binds a person to an undesirable stereotype and directs them [1]. According to the World Health Organization, social stigma in the context of health is defined as a negative relationship between a person or group of people with certain characteristics and a certain disease [2]. Stigma during a pandemic can be defined as a situation in which people are judged by being molded into negative stereotypes, discriminated against, treated differently, and lose status due to a perceived link with the disease. This condition can harm patients, as well as their caregivers, family, friends, and communities, by influencing them through their perceptions, beliefs, and emotions. Stigma can have serious consequences, including fueling fear, anger, and intolerance towards other people. People who are stigmatized are more likely to be reluctant to seek treatment. This may delay disease treatment and increase morbidity and mortality. In addition, feelings of violence or bullying, poor quality of life, disability, increased socioeconomic burden, shame, and self-doubt may occur.

Severe acute respiratory syndrome coronavirus 2 (SARS-CoV-2) is a highly contagious and potentially fatal disease [3]. The rapid global increase in the number of people infected with this virus has increased public fear and anxiety in many regions [4, 5]. This fear and anxiety has resulted in stigma, a new form of discrimination against people affected by COVID-19 in many communities [4].

The aim of the present study was to determine the level of stigmatization in individuals who had COVID-19 with the emergence of the disease and in which direction it subsequently changed, and to determine what can be done in this regard and to make recommendations on issues that can be corrected through health policies.

## Materials and methods

Individuals who presented to Turkey Ankara Medicalpark and Turkey VM Medicalpark Hospitals' Internal Diseases and Pulmonary Diseases polyclinic between May 2021 and April 2022 and who were infected with COVID-19 and recovered were included. The inclusion criteria were as follows: aged 18 years or older, literate, diagnosed with COVID-19 by real-time reverse transcription polymerase chain reaction test and recovered, and infected with COVID-19 in the first six months or the second six months of the pandemic without any symptoms of disease when included in the study. Patients who did not meet these criteria were excluded.

There were COVID-19 restrictions in our country at the time the surveys were filled out. Written informed consent was obtained from the volunteers, and the questionnaire was filled out. The questionnaire was prepared by modification of the questions used for previous infectious diseases [6], each of which examines a separate sociological and psychological condition, together with sociodemographic information. The questionnaire consisted of 29 questions in total, according to a two-point Likert scale, in which the volunteers stated whether they agreed with the statements or not. Before the study, the questionnaire was pre-evaluated by applying it to 20 volunteers who had COVID-19 (10 in the first six months of the pandemic and 10 in the second six months). Written informed consent was also obtained from these 20 volunteers. Any incomprehensible parts or contradictory statements in the questions were changed, and the questionnaire was finalized.

One hundred patients (group 1) who had COVID-19 in the first six months of the pandemic (March to September 2020) and 100 patients (group 2) who had it in the second six months (October 2020 to March 2021) were included in the study. The data of 127 participants who filled out the questionnaire completely and correctly were analyzed.

Ethics committee approval was obtained for the study from the Ankara City Hospital Ethics Committee (number E2-22-1338).

Statistical analysis

The data were evaluated with SPSS v20.0 (IBM Inc., Armonk, New York). Continuous variables were expressed as mean±standard deviation, and categorical variables as numbers (%). The Chi-squared test was used to compare categorical data, and p<0.05 was considered statistically significant.

## Results

Two hundred patients who had been infected with COVID-19 were included in the study. The data of 127 participants who filled out the questionnaire completely and correctly were analyzed. The patients were divided into two groups: those who had the disease in the first six months of the pandemic and those who had it in the second 6 months. The median age of the volunteers was 38 years. Among these patients, 88 patients (69.3%) who filled out the form correctly reported that they had had the disease in the first six months of the pandemic, and 39 patients (30.7%) reported that they had the disease in the second 6 months. Thirty-five of the patients (27.6%) were female and 92 were male (72.4%). When asked "What was your first feeling when you found out about the disease?", 64.6% of the participants reported that they were upset (Table 1).

**Table 1:** Sociodemographic characteristics of the patients PRC - polymerase chain reaction

Characteristics		N (%)
Age (years), median (min-max)		38 (17-73)
Sex	Female	35 (27.6%)
	Male	92 (72.4%)
Education	Elementary school	17 (12.4%)
	High school	66 (52.0%)
	University	35 (27.6%)
	Master's degree	9 (7.1%)
Marital status	Single	15 (11.8%)
	Married	112 (88.2%)
Has children	No	78 (61.4%)
	Yes	49 (38.6%)
Occupation	Teacher	6 (4.7%)
	Manager	72 (56.7%)
	Employee	13 (10.2%)
	Housewife	15 (11.8%)
	Government official	5 (3.9%)
	Health professional	4 (3.1%)
	Artisan	4 (3.1%)
	Retired	5 (3.9%)
	Student	3 (2.4%)
Economic situation	Good	73 (57.5%)
	Moderate	52 (40.9%)
	Bad	2 (1.6%)
Blood group	O rh+	78 (61.4%)
	O rh-	1 (0.8%)
	A rh+	28 (22.0%)
	A rh-	2 (1.6%)
	B rh+	6 (4.7%)
	B rh-	3 (2.4%)
	AB rh+	8 (6.3%)
	AB rh-	1 (0.8%)
Disease history	Had the disease in the first six months of the pandemic	88 (69.3%)
	Had the disease after the first six months of the pandemic	39 (30.7%)
PCR results	Negative	3 (2.4%)
	Positive	124 (97.6%)
Hospitalization	No	108 (85.0%)
	Yes	19 (15.0%)
Family members with concurrent disease	Single	101 (79.5%)
	Mother	6 (4.7%)
	Father	4 (3.1%)
	Mother and father	4 (3.1%)
	Mother, father, and sibling	6 (4.7%)
	Sibling	3 (2.4%)
	Index case and wife/husband	2 (1.6%)
	All family	1 (0.8%)
Is there a member with COVID-19 in the family?	No	54 (42.5%)
	Yes	73 (57.5%)
Could you tell everyone that you were infected with COVID-19?	No	18 (14,2%)
	Yes	109 (85.8%)
What was your first feeling when you found out about the disease?	Sad	82 (64.6%)
	Refused to accept	6 (4.7%)
	Connected it to fate	11 (8.7%)
	Did not care	10 (7.9%)
	Got angry	7 (5.5%)
	Other	11 (8.7%)

In response to the questionnaire statement "I could not easily tell my social circle that I had caught COVID-19", 69.3% of the participants in the first group and 100% in the second group stated that they did not agree (p=0.001). Further, 39.8% of the participants in the first group and 93.3% in the second group reported that they did not agree with the statement "When I found out I had COVID-19, I didn't find pleasure in anything" (p<0.001). Another question asked to the participants was whether they agreed with the statement "People often think that those with poor hygiene habits get COVID-19"; 63.6% in the first group and 7.7% in the second group agreed with this (p=0.001). Some 76.1% of the participants in the first group and 94.9% in the second group did not agree with the statement "I thought COVID-19 was a punishment for me" (p=0.011). Fifty percent of the participants in the first group and 94.9% in the second group did not agree with the statement "My relatives were acting like it was my fault that I got COVID-19" (p<0.001). Moreover, 56.8% of the participants in the first group and 97.4% in the second group reported that they did not agree with the statement "Employers may terminate the employment of employees who they find out have contracted COVID-19" (p<0.001). Some 66.3% of the participants in the first group and 92.3% in the second group did not agree with the statement "Because I caught COVID-19, I considered myself as someone who was constantly infecting others" (p=0.001). Further, 53.4% of the participants in the first group and 87.2% in the second group stated that they did not agree with the statement "Catching COVID-19 made me feel lonely" (p<0.001). In addition, 60.2% of the participants in the first group and 10.3% in the second group stated that they agreed with the statement "People did not want to work in the same environment (in the same room) with someone who had contracted and recovered from COVID-19" (p<0.001). It was seen that 72.7% of the participants in the first group and 87.2% in the second group agreed with the statement "Telling my friends that I caught COVID-19 did not spoil my relations with them" (p=0.024). It was found that 63.6% of the participants in the first group and 35.9% in the second group agreed with the statement "At the time when I caught COVID-19, people excluded patients from society" (p=0.004). Finally, 80.7% of the participants in the first group and 38.5% in the second group agreed with the statement "There was social discrimination against people who caught COVID-19" (p<0.001) (Table 2).

**Table 2:** Responses to the questionnaire of those who had the disease in the first six months and six months after the COVID-19 pandemic

Propositions	Answers	Group 1 <6 months	Group 2 ≥6 months	p-value
1. I could not easily tell my social circle that I had caught COVID-19.	I agree	26 (29.5%)	0 (0%)	0.001*
	I do not agree	61 (69.3%)	39 (100%)	
2. I wasn't blaming myself for catching COVID-19.	I agree	69 (78.4%)	26 (66.7%)	0.016
	I do not agree	19 (21.6%)	13 (33.3%)	
3. When I found out I had COVID-19, I didn't find pleasure in anything.	I agree	53 (60.2%)	3 (7.7%)	0.000*
	I do not agree	35 (39.8%)	36 (93.3%)	
4. People don't hesitate to marry someone who has had COVID-19.	I agree	70 (79.5%)	26 (66.7%)	0.119
	I do not agree	18 (20.5%)	13 (33.3%)	
5. People often think that those with poor hygiene habits get COVID-19.	I agree	56 (63.6%)	12 (7.7%)	0.001*
	I do not agree	32 (36.4%)	24 (17.9%)	
6. I was ashamed to have COVID-19.	I agree	19 (21.6%)	2 (5.1%)	0.021
	I do not agree	69 (78.4%)	37 (94.9%)	
7. I thought COVID-19 was a punishment for me.	I agree	21 (23.9%)	2 (5.1%)	0.011*
	I do not agree	67 (76.1)	37 (94.9%)	
8. My relatives were acting like it was my fault that I got COVID-19.	I agree	44 (50%)	2 (5.1%)	0.000*
	I do not agree	44 (50%)	37 (94.9%)	
9. My friends have not greeted me since I caught COVID-19.	I agree	20 (22.7%)	4 (10.3%)	0.098
	I do not agree	68 (77.3%)	35 (89.7%)	
10. When I contracted COVID-19 and during the early recovery period, I was avoiding social situations.	I agree	57 (64.8%)	30 (76.9%)	0.174
	I do not agree	31 (35.2%)	9 (23.1%)	
11. I was physically distant from people in the early period when I caught/recovered from COVID-19.	I agree	76 (86.4%)	30 (76.9%)	0.263
	I do not agree	12 (13.6%)	9 (23.1%)	
12. Employers may terminate the employment of employees who they find out have contracted COVID-19.	I agree	38 (43.2%)	1 (2.6%)	0.000*
	I do not agree	50 (56.8%)	38 (97.4%)	
13. Because I caught COVID-19, I saw myself as someone who was constantly infecting others.	I agree	32 (36.4%)	3 (7.7%)	0.001*
	I do not agree	56 (63.6%)	36 (92.3%)	
14. Catching COVID-19 made me feel lonely.	I agree	41 (46.6%)	5 (12.8%)	0.000*
	I do not agree	47 (53.4%)	34 (87.2%)	
15. After contracting COVID-19 and recovering, I felt as good as other people.	I agree	51 (58%)	28 (71.8%)	0.138
	I do not agree	37 (32%)	18 (28.2%)	
16. My relatives continued to meet with me following the rules (mask, distance, and hygiene) when I caught COVID-19 and during the early recovery period.	I agree	65 (73.9%)	27 (69.2%)	0.590
	I do not agree	23 (26.1%)	12 (30.8%)	
17. Sometime after my friends got over COVID-19, they were eating what I prepared/served when they came to visit me.	I agree	46 (52.3%)	24 (61.5%)	0.333
	I do not agree	42 (47.7%)	15 (38.5%)	
18. After I had COVID-19 and recovered, I thought that I was spreading the microbe around me, and I stayed away from my social circle.	I agree	80 (90.9%)	31 (79.5%)	0.074
	I do not agree	8 (9.1%)	8 (20.5%)	
19. After I had COVID-19 and got better, I was constantly staying in my room so that my family would not get sick.	I agree	74 (84.1%)	28 (73.7%)	0.172
	I do not agree	14 (15.9%)	10 (26.3%)	
20. When I had COVID-19 and recovered, I could comfortably drink something (tea, coffee, soft drink) in social environments.	I agree	68 (77.3%)	30 (76.9%)	0.965
	I do not agree	20 (22.7%)	9 (31.6%)	
21. When people learned that I had contracted COVID-19 and recovered, they wouldn't mind me being close to them and their children.	I agree	69 (78.4%)	27 (69.2%)	0.267
	I do not agree	19 (21.6%)	12 (30.8%)	
22. During my illness, people did not want to work in the same environment (in the same room) as someone who had contracted and recovered from COVID-19.	I agree	53 (60.2%)	4 (10.3%)	0.000*
	I do not agree	35 (39.8%)	35 (89.7%)	
23. When I had COVID-19, people used to think that getting COVID-19 would not affect the marriage or relationship.	I agree	73 (83%)	31 (79.5%)	0.640
	I do not agree	15 (17%)	8 (20.5%)	
24. During the time I caught COVID-19, my illness did not affect my relations with my mail.	I agree	81 (92%)	36 (92.3%)	0.960
	I do not agree	7 (8%)	3 (7.7%)	
25. Telling my friends that I caught COVID-19 did not spoil my relations with them.	I agree	64 (72.7%)	34 (87.2%)	0.024
	I do not agree	24 (27.3%)	5 (12.8%)	
26. At the time when I caught COVID-19, people excluded patients from society.	I agree	56 (63.6%)	14 (35.9%)	0.004*
	I do not agree	32 (36.4%)	25 (64.1%)	
27. People would not hesitate to travel on the same bus as someone who has contracted and recovered from COVID-19.	I agree	64 (72.7%)	26 (66.7%)	0.488
	I do not agree	24 (27.3%)	13 (33.3%)	
28. The daughter-in-law or son-in-law of a person whose mother-in-law/father-in-law has caught COVID-19 would not take the trouble to look after him or her in their own home.	I agree	56 (63.6%)	18 (46.2%)	0.065
	I do not agree	32 (36.4%)	21 (53.8%)	
29. There was social discrimination against people who caught COVID-19.	I agree	65 (73.9%)	15 (38.5%)	0.000*
	I do not agree	23 (26.1%)	24 (61.5%)	

## Discussion

Stigmatization is a situation that has been encountered frequently in the past during pandemics and resulted in discrimination against these patient groups and negative social consequences [7]. Epidemics and pandemics are times when people are judged, discriminated against, treated separately, and/or lost status due to their illness. Having a contagious disease and receiving treatment for this disease can adversely affect patients and their environment (caregiver, family, friends, etc.). A meta-analysis found a prevalence of stigma of 34% in all populations during infectious disease outbreaks (e.g., SARS, H1N1, Zika, and COVID-19) [8]. However, as people's knowledge and experiences related to the disease increase, prejudices against it decrease, and they can display more rational approaches. To the best of our knowledge, this is the first study in the literature to investigate and demonstrate the reduction in stigma as time progresses during the pandemic. In a study conducted in China, including 4119 participants, it was shown that COVID-19 had negative effects on loneliness, depression, and stigma [9].

Since SARS-CoV-2/COVID-19 is an airborne disease, it requires adherence to certain rules for social cohesion and social isolation. This makes people hide their illness and, therefore, delay diagnosis and treatment in order to avoid discrimination. Stigma can weaken the country's social matrix and become a barrier to disease control. COVID-19 has led to stigma and discrimination among various groups of people in different populations. Healthcare workers caring for those affected by COVID-19, those who have had COVID-19, those belonging to lower socioeconomic groups, and those with certain religious and racial identities are the most vulnerable to discrimination [10]. During the 2003 SARS epidemic, 20-49% of healthcare professionals caring for SARS patients were stigmatized over fear of transmitting the disease by people in their community, according to studies [11, 12]. The families of healthcare workers also faced such discrimination [11].

In the present study, it was discussed whether the level of stigma associated with COVID-19 in Turkish society changed during the first six months and the second six months. In order to explore this situation, the participants were presented with the statement "There was social discrimination against people who caught COVID-19". They were asked whether they agreed with this. A comparison of the answers revealed that 73.9% of the participants in the first group and 38.5% in the second group agreed. In addition, they were asked whether they agreed that "At the time when I caught COVID-19, people excluded patients from society". Their answers showed that 63.6% of the participants in the first group and 35.9% in the second group agreed, which indicated that stigma-related discrimination decreases with increasing recognition of the disease and increasing medical experience about it. Regarding the statement "I cannot easily tell my social circle that I have caught COVID-19", 69.3% of the participants in the first group and 100% in the second group did not agree. This result showed that stigmatization can be prevented by supplying correct information to the public. During a pandemic, stigma decreases with the provision of this information. The systematic review and meta-analysis study by Yuan et al. [8] and the study by Islam et al. [13] showed that a one-point increase in basic knowledge was associated with a 0.51-point decrease in the core stigma index.

In order to assess people's status and anxiety about losing their job during the pandemic, the participants were told, "Employers may terminate the employment of employees who they find out have contracted COVID-19". They were asked whether they agreed with this. According to their answers, 56.8% of the participants in the first group and 97.4% in the second group did not agree. The responses given to this statement indicated that at the beginning of the epidemic, the participants had serious concerns about losing their status and job, but this anxiety decreased over time.

COVID-19 has been accepted by the public as a dangerous and frightening infectious disease in a short time. This leads to an inevitable feeling of embarrassment for both the patients and their families, leading to social isolation and a tendency towards secrecy regarding the diagnosis [14]. Individuals with a high level of social support describe themselves as luckier and more positive than others. A positive benefit of perceived social support is that patients can believe that others will be there to help them when needed, enabling individuals to cope with life stressors more effectively [15]. Therefore, social support from family and friends can be helpful in cases of stigma [16]. In order to investigate this situation, the participants were asked if they agreed with the statement "My relatives were acting like it was my fault that I got COVID-19". Fifty percent of the participants in the first group and 94.9% in the second group did not agree. This showed that the level of stigma within the family decreased over time. The participants were asked whether they agreed with the statement "Catching COVID-19 will not affect my relationships with my family". When the answers given to this statement were compared, 92% of the participants in the first group and 92.3% in the second group agreed. The situation suggests that in this country, family members are less likely to be stigmatized and excluded by other family members, who are more likely to get COVID-19 due to the sick individual. Supporting this result, a study involving 1076 participants showed that stigma decreased with strong social and family ties [17].

Evidence emerging from many parts of Sub-Saharan Africa shows that those who have recovered, as well as their families and other closely related persons, experience extreme stigma [18]. Such behaviors and actions not only disrupt social cohesion but also disrupt the measures taken against COVID-19. Lessons learned from previous epidemics such as Middle East respiratory syndrome coronavirus (MERS-CoV), HIV, and Ebola show that stigma tends to persist during and after the epidemic [19-21]. Various media reports document that people suspected of having COVID-19 have been kicked out of their rental apartments and removed from their workplaces, especially after completing the mandatory quarantine [22]. When the participants were asked whether they agreed with the statement "People do not want to work in the same room with someone who has contracted COVID-19 and recovered", 60.2% of the participants in the first group and 10.3% in the second group agreed. This may be because people have experienced this situation due to the prolongation of the pandemic, the continuation of social life, and the necessity of returning to business life.

Previous studies focusing on people with mental illness [23], epilepsy [24], or HIV infection [25] show that social support is inversely related to perceived stigma. People who are stigmatized may become withdrawn over time and feel lonely. They may feel that they are not part of the society they live in. They may experience anxiety, hopelessness, helplessness, and pessimism. This can pave the way for mental disorders. They may think that they deserve this, may experience intense anger, and may display harmful behaviors toward themselves and those around them. Due to the stigma associated with COVID-19, the psychological problems of those affected may worsen, and individuals discharged from quarantine and isolation experience serious negative effects on their mental health due to stigma and consequent emotional confusion [14, 26]. To explore this situation, the participants were asked whether they agreed with the following statement: "Catching COVID-19 made me feel lonely"; 53.4% of the participants in the first group and 87.2% of the participants in the second group did not agree. In addition, to investigate this issue, the participants were asked whether they agreed with the statement "When I found out I had COVID-19, I didn't find pleasure in anything". 39.8% of the participants in the first group and 93.3% of those in the second group did not agree with this statement. There was a statistically significant difference between the two groups in the responses given to both statements. This suggests that the effect of the pandemic on individuals has decreased over time and a state of obligatory acceptance of pandemic conditions has developed.

Due to the rapid spread of the virus, recovering COVID-19 patients may have had to stay away from family members, close relatives, and friends and practice social distancing [14]. In our study, we asked the participants whether they agreed with the statements "After catching COVID-19, I was staying away from my close circle so as not to spread the microbe to those around me" and "After contracting COVID-19, I was constantly staying in my room so that my family would not get sick". Responses to the two statements were similar between the groups. The answers given show the fear and anxiety of individuals about harming their family members and others related to the disease throughout the pandemic.

People who have been infected and recovered may have unfounded fears of contracting the disease through personal contact with their friends and circles and may develop anger, frustration, and sadness due to damaged relationships [14]. In order to explore this issue, the participants were asked whether they agreed with the statement "Because I caught COVID-19, I considered myself as someone who was constantly infecting others" and "Telling my friends that I have COVID-19 does not spoil my relationship with them". When the answers to the first question were compared, 66.3% of the participants in group 1 and 92.3% of those in the second group did not agree. When the answers to the second question were compared, 72.7% of the participants in the first group and 87.2% of those in the second group did not agree. There was a statistically significant difference between the two groups. This may suggest how important the concern and knowledge level of individuals about the spread of contact-related disease during an epidemic is. In addition, the participants were asked whether they agreed with the statement "My friends have not greeted me since I caught COVID-19". There was no difference in the answers given between the two groups. We think that this is due to the cultural structure of our society regarding friendship and commitment.

Considering that COVID-19 particularly affects the population over the age of 65, it is known that this group is at high risk of stigma [27]. In order to evaluate the elderly population exposed to COVID-19 from the perspective of others, the participants were asked whether they agreed with the statement "The daughter-in-law or son-in-law of a person whose mother-in-law/father-in-law has caught COVID-19 would not take the trouble to look after him/her in their own home". A comparison of the answers showed that 63.6% of the participants in the first group and 46.2% of those in the second group agreed. The difference was statistically significant. This shows that social and cultural values may be a factor that prevents stigmatization.

The implementation of travel bans, movement restrictions, and quarantines during the pandemic may have disproportionately affected people already stigmatized, including the homeless, incarcerated persons, immigrants and refugees, undocumented immigrants, and minorities [28, 29]. They were asked whether they agreed with the statement "People who do not comply think they have caught COVID-19"; 80.7% of the participants in the first group and 38.5% of those in the second group agreed. There was a statistically significant difference between the groups. This shows that the exaggerated theories about disease transmission, which emerged with the onset of the pandemic, have been replaced by a decrease in this idea after the recognition of the disease and learning the correct prevention methods.

There were some limitations of our study. The most important limitation is that patients who had experienced COVID-19 in the first or second six months were asked to respond by considering that period. We think that these patients may not have been able to give answers that fully reflect this period since it has been a while since they experienced the stigma experienced during their illness. Moreover, the number of participants was relatively small. The number of patients could have been increased by adding more centers. Another important limitation was that the validity and reliability of the questionnaire administered to the patients in the study could not be evaluated in a large patient population.

## Conclusions

Consequently, stigma and the resulting discrimination during the pandemic can pose significant challenges in protecting the health of people in general and the vulnerable and can reduce the effectiveness of public health measures implemented to contain the spread of the disease. The exacerbation of existing social inequalities and the formation of new forms of social division may lead to the deterioration of human relations. In the present study, it is obvious that stigma decreased in the later stages of the COVID-19 pandemic, and the social and societal negative effects of those exposed to stigma also decreased over time. The lack of information about the disease and the fact that the public did not know anything about the disease were important in this regard. Conducting this study, which evaluated stigma during the pandemic, in larger patient populations, including different races, may help us to obtain generalizable information. In possible pandemic situations, stigma can be combated with increasing importance given to public information.
